# Supplementary material for: Heparan Sulfate Proteoglycans as Potential Markers for In Vitro Human Neural Lineage Specification
Source: Cells. 2025 Jul 26;14(15):1158. doi: 10.3390/cells14151158 (PMC12345897; doi:10.3390/cells14151158)
Supplement: Supplementary file 1 [file cells-14-01158-s001.zip › Supplementary Information_Online Resource Captions.pdf]

Heparan Sulfate Proteoglycans as Markers in Human Neural Progenitor Cell Line Short-Term Lineage Differentiation

Journal: Molecular Neurobiology

Chieh Yu[1,*], Ian W Peall[1,3], Son H Pham[1], Duy LB Nguyen[1,3], Lyn R Griffiths[1], Rachel K Okolicsanyi[1,3], Larisa M Haupt[1,2, 3**].

[1]Centre for Genomics & Personalised Health, Genomics Research Centre, School of Biomedical Sciences, Queensland University of Technology, Brisbane, Australia, [2]ARC Training Centre for Cell and Tissue Engineering Technologies, Queensland University of Technology (QUT), Australia, [3]Max Planck Queensland Centre for the Materials Sciences of Extracellular Matrices

[*]Present Address: Department of Cell and Tissue Biology, University of California, San Francisco, USA

[**]Corresponding author

Associate Professor Larisa Haupt

Centre for Genomics & Personalised Health, Genomics Research Centre

Queensland University of Technology

60 Musk Avenue, Kelvin Grove QLD 4059

Australia

Telephone: +617 3138 0801

Fax: +617 3138 6039

E-mail: larisa.haupt@qut.edu.au

Supplementary Information

Online Resources

ESM_1. Positive Control – hNSC H9 D60 neuronal culture

ESM_2. Negative Control – MCF-7 Human breast cancer cell line

ESM_3. SH-SY5Y Neuronal Differentiation D18

ESM_4. ReNcell CX Astrocyte Differentiation D14

ESM_5. ReNcell VM Mixed Neural Culture D14

ESM_6. ReNcell VM +BDNF Culture D14

ESM_7. ReNcell VM +PDGF Culture D14
