# Supplementary figures and images for: Heparan Sulfate Proteoglycans as Potential Markers for In Vitro Human Neural Lineage Specification
Source: Cells. 2025 Jul 26;14(15):1158. doi: 10.3390/cells14151158 (PMC12345897; doi:10.3390/cells14151158)

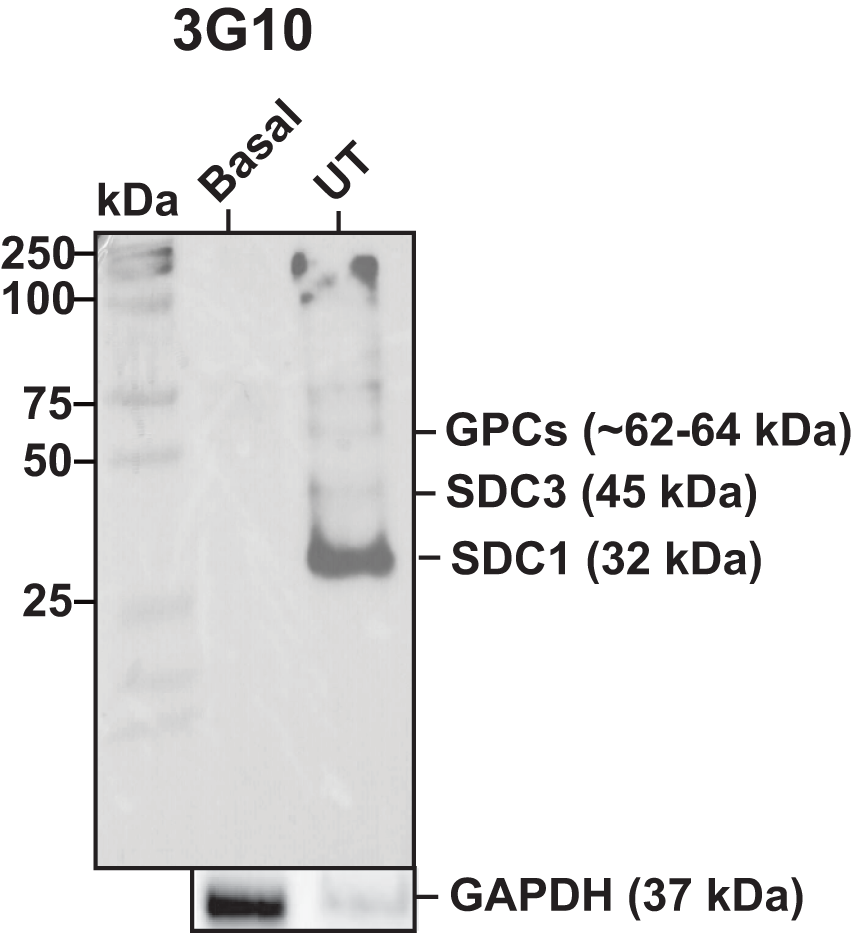

Supplement: Supplementary file 1 [file cells-14-01158-s001.zip › Yu et al. Suplementary Figure S3.png]

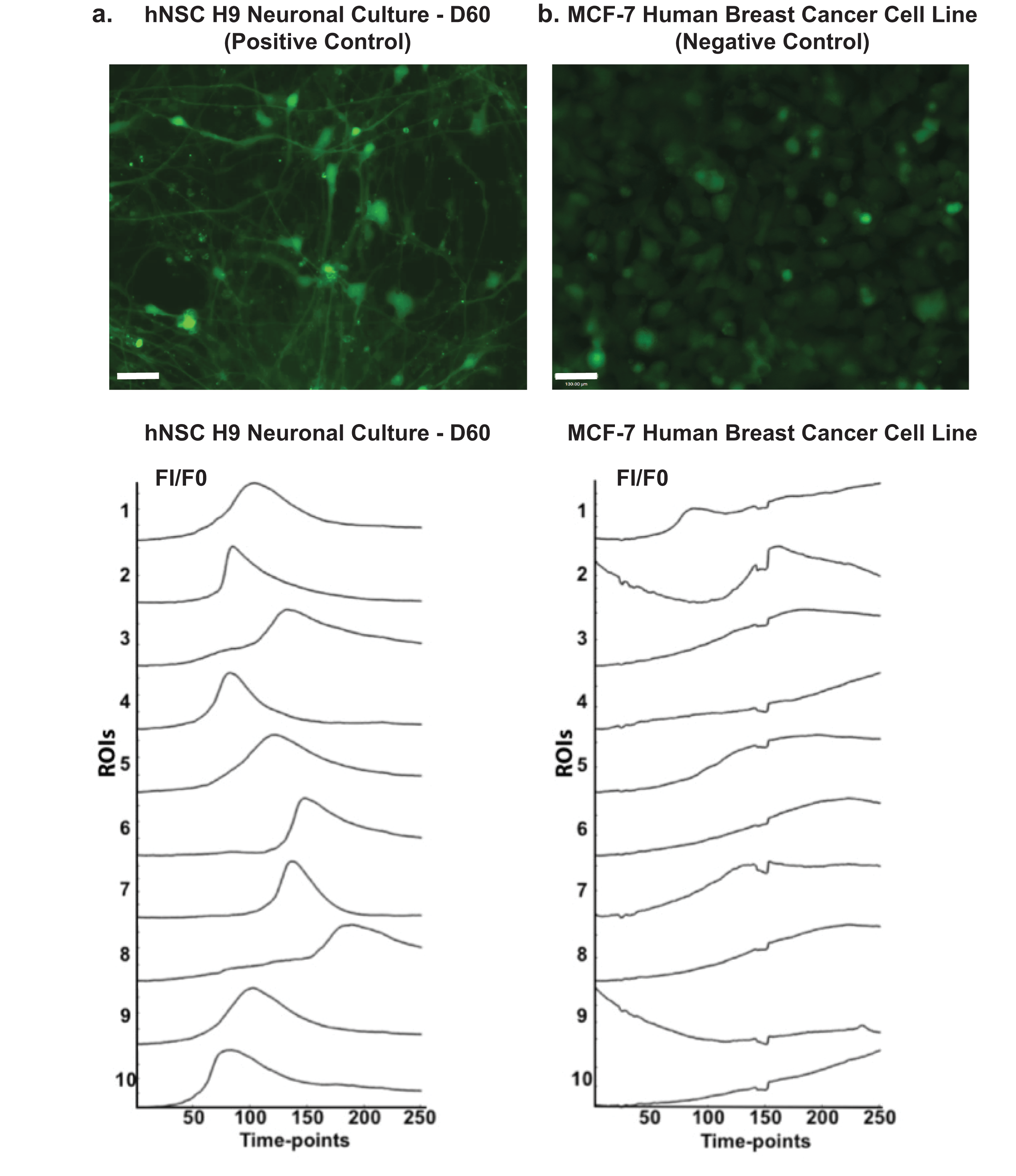

Supplement: Supplementary file 1 [file cells-14-01158-s001.zip › Yu et al. Supplementary Figure S1.png]

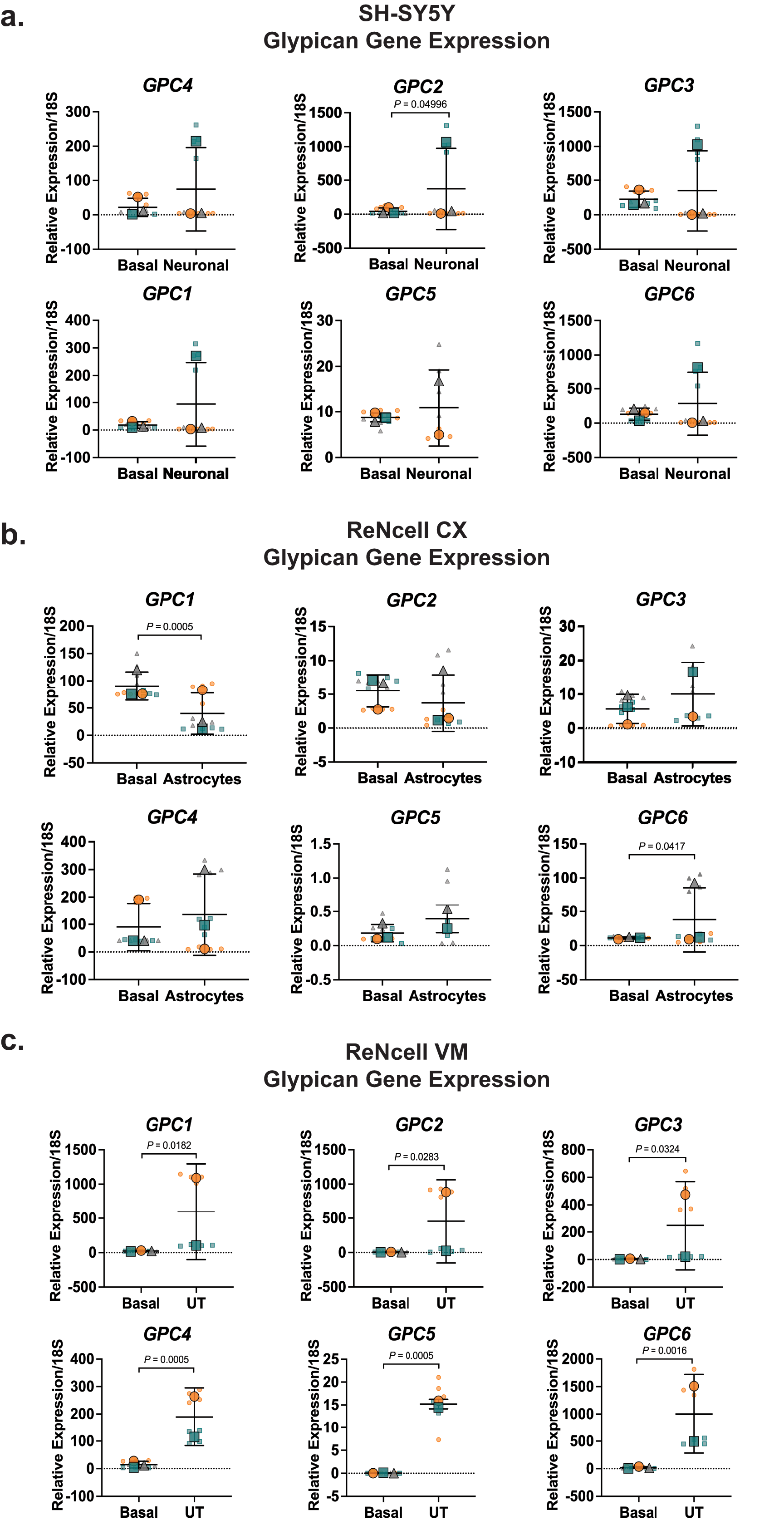

Supplement: Supplementary file 1 [file cells-14-01158-s001.zip › Yu et al. Supplementary Figure S2.png]
